# Influenza: a scientometric and density-equalizing analysis

**DOI:** 10.1186/1471-2334-13-454

**Published:** 2013-09-30

**Authors:** Ralph Fricke, Stefanie Uibel, Doris Klingelhoefer, David A Groneberg

**Affiliations:** 1 Institute of Occupational Medicine, Social Medicine and Environmental Medicine, Goethe-University, Frankfurt, Germany

## Abstract

**Background:**

Novel influenza in 2009 caused by H1N1, as well as the seasonal influenza, still are a challenge for the public health sectors worldwide. An increasing number of publications referring to this infectious disease make it difficult to distinguish relevant research output. The current study used scientometric indices for a detailed investigation on influenza related research activity and the method of density equalizing mapping to make the differences of the overall research worldwide obvious. The aim of the study was to compare scientific effort over the time as well as geographical distribution including the cooperation on national and international level.

**Methods:**

Therefore, publication data was retrieved from *Web of Science* (WoS) of Thomson Scientific. Subsequently the data was analysed in order to show geographical distributions and the development of the research output over the time.

The query retrieved 51,418 publications that are listed in WoS for the time interval from 1900 to 2009. There is a continuous increase in research output and general citation activity especially since 1990.

**Results:**

The identified all in all 51,418 publications were published by researchers from 151 different countries. Scientists from the USA participate in more than 37 percent of all publications, followed by researchers from the UK and Germany with more than five percent. In addition, the USA is in the focus of international cooperation.

In terms of number of publications on influenza, the *Journal of Virology* ranks first, followed by *Vaccine* and *Virology*. The highest impact factor (IF 2009) in this selection can be established for *The Lancet* (30.75). *Robert Webster* seems to be the most prolific author contributing the most publications in the field of influenza.

**Conclusions:**

This study reveals an increasing and wide research interest in influenza. Nevertheless, citation based-declaration of scientific quality should be considered critically due to distortion by self-citation and co-authorship.

## Background

Zoonotic emergence of human infectious diseases leads to a global risk to public health. The Spanish flu in 1918/19, most probably originated in birds, caused approx. 50 million deaths worldwide [1]. The experience of H5N1, with a case fatality rate of at least 50 percent in 2003, the outbreak of the “novel influenza” (H1N1) in 2009 caused concerns of a global spread of this pandemic with millions of deaths estimated. Fortunately, the H1N1 pandemic was less severe compared to earlier pandemics. Nevertheless, seasonal influenza is still a challenge to local public health sectors all over the world [2,3]. In recent years, enormous efforts have been made to improve diagnostic and therapeutic methods. However, problems in prevention and control of influenza persisted due to rising urbanization and mobility [4,5]. The global threat of an influenza pandemic is also a challenge for international and interdisciplinary cooperation. Since the first available publications at the end of the 19^th^ century, the amount of publication dealing with influenza is substantially and continuously increasing. The vast amount of publications referring to influenza and the difficulties in handling disease once it emerged, illustrates the need of a sound evaluation of the scientific output. Therefore, the present study investigates research accomplishments and their distribution using scientometrics and density equalizing mapping in combination.

## Methods

### Data sources

Data was retrieved from the database *Web of Science* (WoS) by Thomson Scientific and, for further specific analyses, in the Medline database (*PubMed*) by the U.S. National Library of Medicine. Additional data was interrogated from OECD Statistics (Gross domestic product (GDP), spending for research and development (R&D), number of researchers as full-time equivalent per thousand people employed) [6].

### Search strategy

“Influenza”, “Flu” and “Grippe” was entered in the search field and combined with the Boolean operator “OR” to retrieve the overall number of items. Further investigations used the “Analyze Results” function and the “Citation Report” provided by the WoS database.

### Timeframe

The timeframe covers the years from 1900 to 2009. Results from 2010 to 2012 were not considered due to incomplete data acquisition at the time of data query.

### Data analysis

Items retrieved from the data query in WoS were analysed by publication date, country of origin, source title, author and institution. Findings were transferred to Excel charts and pictured in diagrams. Furthermore, all published items were analysed with the function “citation report”. For all publications referring to influenza, the total number of citations and the average citation per item (citation rate) were calculated. In this regard, the number of publications can be seen as an indicator of quantity of the research productivity. Citation analysis is used as an indicator for the impact of publications for the scientific community, and, in consideration of methodical limitations, for research quality. Country related data were presented by using density equalizing mapping procedures based on Gastner’s and Newman’s algorithm [7]. These were used as described in previous studies [8]. Using this approach, the size of territories (e.g. countries) was aligned in correspondence to the peculiarity of selected variables (e.g. the number of publications, the number of citations and the average citation per country). The resulting maps are thus distorted depending on the chosen parameter. Finally, cooperation between countries and institutions was investigated. A journal contribution published by at least two authors coming from different countries or institutions is defined as cooperation. Using a specially developed software module, the values for the cooperation of each pair of countries were visualized as vectors. The thickness and colour of a vector elucidates the number of international and institutional cooperation, respectively [9]. To ensure legibility, a minimum threshold of ten cooperative activities was defined.

## Results

### Amount of published items and citation behaviour

The query in the WoS database results in 51,418 publications with a reference to influenza. In the research timeframe, a continuous and accelerating increase of publications in this subject area can be determined, especially in the last decade. A first remarkable increase in influenza related research output in WoS can be noticed in 1918 to 1921. Almost half of all publications (48.7 percent) were published in the last ten years of the examined period (2000–2009). The most citations were found in 1998. A citation rate (CR) of more than 30 can be shown in 1942 and 1944 as well as 1991 to 2000.

### Analysis of research origin and cooperation

**Figure 1:**
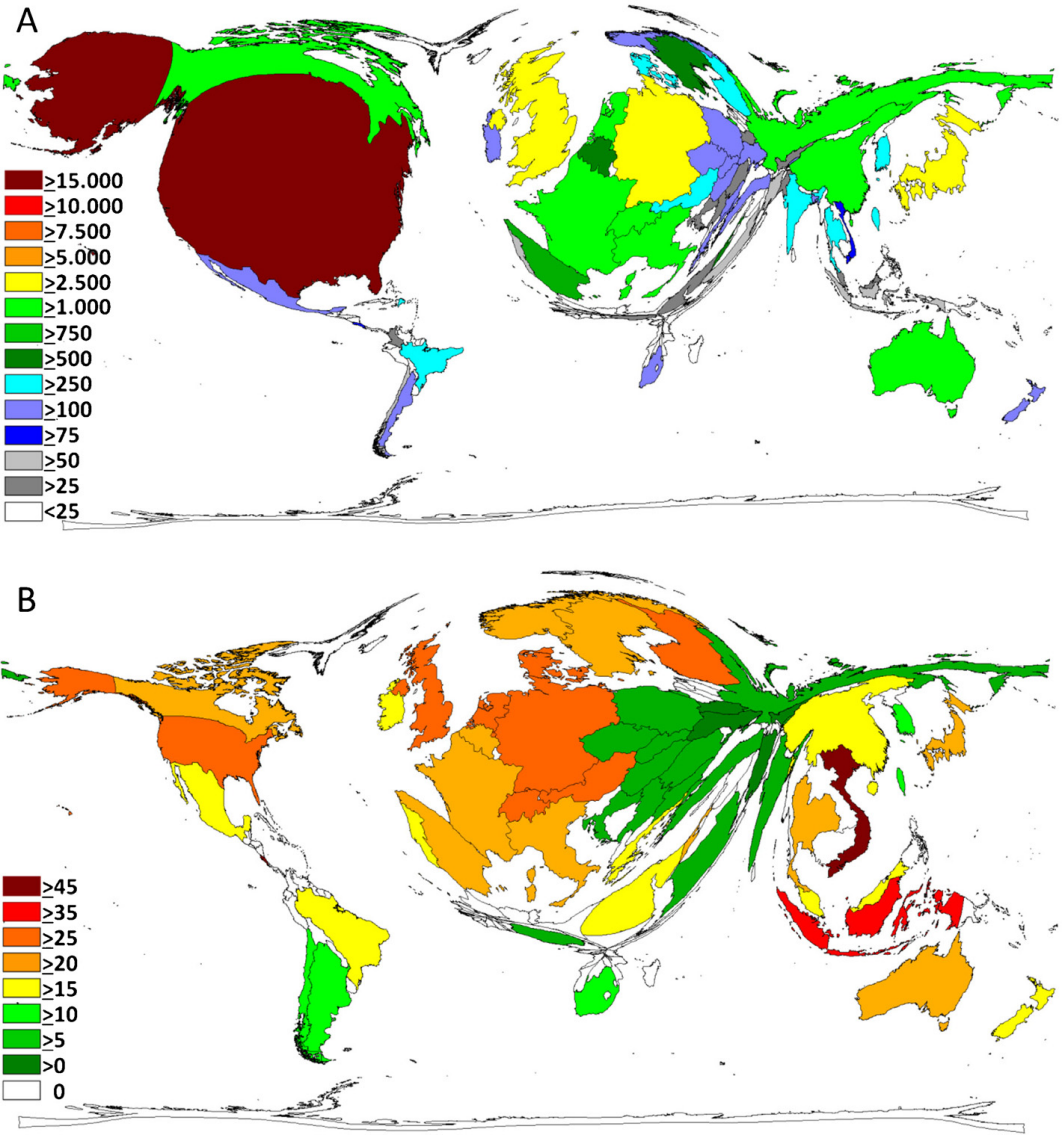
**Density equalizing calculations. (A)** Map illustrating the total number of influenza related publications for each country for the period 1900–2009, Legend: Number of publications. **(B)** Map showing average citation per item for each country for the period 1900–2009 (threshold > 30 publications), Legend: Average number of citations.

**Figure 2:**
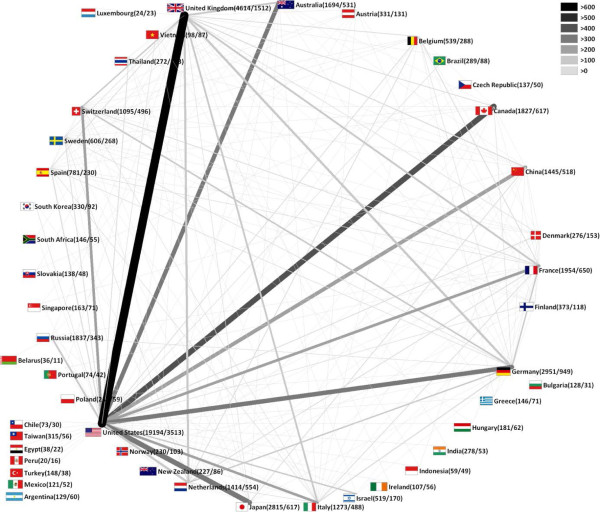
**Country network analysis.** Threshold excludes cooperation with less than 10 items, numbers in brackets: number of publications/cooperation articles, width and colour of beams: frequency of cooperation.

The 51,418 articles were published by 151 different countries. Researchers from the United States of America (USA) participated in more than 37 percent of all publications referring to influenza (19,194) followed by scientists from the United Kingdom (UK) (4,614), Germany (2,951) and Japan (2,815). Furthermore, there are eight countries with more than 1,000 published items (Table 1). Accordingly, these countries dominate the cartogram, illustrating the research output by territory (Figure 1A). In contrast, major parts of South America, Africa and Asia are minimized. As expected, the publications from the USA receive most citations of all (588,474), followed by the publications from the UK, Germany and Japan. While Vietnam and Indonesia only published 98 and 59 articles respectively, they appear in a leading position when considering the average citation rate per country (Vietnam 49, Indonesia 35) (Figure 1B). Those articles, focusing on HPAI/H5N1, were mainly published between 2004 and 2009. Looking at the analysis of cooperation, most efforts are located in the USA with the most productive cooperation between the USA and the UK (646), the USA and Canada (421), the USA and Japan (385), the USA and Germany (350) and between the USA and Australia (324) (Figure 2).

**Table 1:** Publications, citations and citation rates sorted by countries

**Country**	**Published items**	**Total citations**	**Citation rate**
United States	19,194	588,474	30.66
United Kingdom	4,614	143,792	31.16
Germany	2,951	82,184	27.84
Japan	2,815	56,787	20.17
France	1,954	41,167	21.07
Russia	1,837	12,493	6.80
Canada	1,827	38,181	20.90
Australia	1,694	41,937	24.76
China	1,445	27,899	19.30
Netherlands	1,414	35,535	25.13
Italy	1,273	26,995	21.21
Switzerland	1,095	33,212	30.33

### Institutional analysis and cooperation

**Figure 3:**
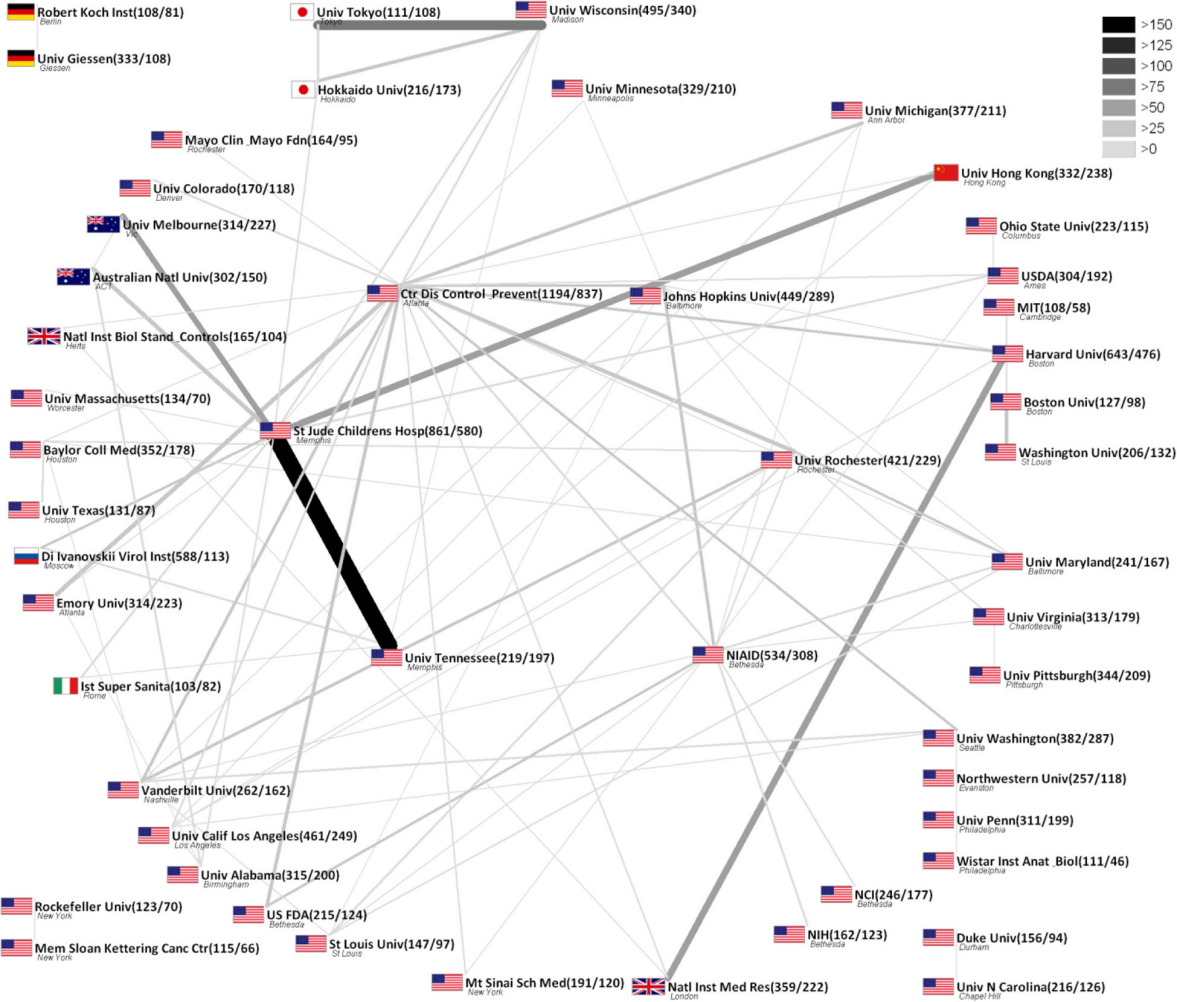
**Institution network analysis.** Threshold > 100 publications, > 10 cooperation, numbers in brackets: number of publications/cooperation articles, width and colour of beams: frequency of cooperation.

A total number of 24,709 institutions participated in publications dealing with influenza in the given research timeframe. The majority of institutions originate from the USA (5,218) analogous to the USA being also the most productive country. UK (1,959), Japan (1,919), France (1,918) and Germany (1,848) also provide a high frequency of scientific institutions. Overall, the *Centers for Disease Control and Prevention* (CDC) ranks first (1,194) followed by the *St. Jude Children’s Hospital* (861) and *Harvard University* (643). The centre of institutional cooperation is the USA as most of cooperating institutions originate there. The most intense cooperation takes place between the *University of Tennessee* and the *St. Jude Children’s Hospital* with 165 articles published in cooperation. Leading international cooperation exists between the *University of Wisconsin* and *Tokyo University* (97 common articles) (Figure 3).

### Research infrastructure in selected countries

Research infrastructure plays an important role in controlling infectious diseases. Identification and spread of Influenza virus and development of suitable vaccine are examples for the need of sufficient research networking. In this study we choose the USA and the European Union as comparable economic areas on the one hand. On the other hand China and Russia were also included in the analysis.

The USA spends 2.64 percent of its Gross Domestic Product (GDP) for research and development (R&D), followed by the European Union (EU-27) with 1.75 percent, China (1.19 percent) and Russia (1.13 percent). Considering the GDP itself, the USA spends the most funds for R&D by far. Looking at the number of researchers (per thousand employees), the USA ranks first as well followed by the EU. Russia ranks last with economic efforts for R&D and annual number of published items (Table 2). Looking at the data in more detail, an increasing expenditure on R&D for the USA, the EU-27 and in particular China can be shown. China has almost tripled its GDP during 2000–2008 and additionally the expenditure on R&D rises from 0.9 to 1.4 percent of GDP. In contrast, for Russia, decreasing spending for R&D can be shown for the last four years.

**Table 2:** GDP (in Bill. $-Dollar), R&D in percent of GDP, Researcher (full-time equivalent per thousand people employed) and published items; average 2000-2008

	**GDP**	**R&D**	**Researchers**	**Published items**
	**2000–2008**	**2000–2008**	**2000–2008**	**2000–2008**
USA	11991.17	2.64	9.69	1000.11
EU-27	12808.28	1.75	5.89	752.44
China	5043.44	1.19	1.30	104.67
Russia	1555.22	1.13	7.13	30.11

### Journal analysis

**Figure 4:**
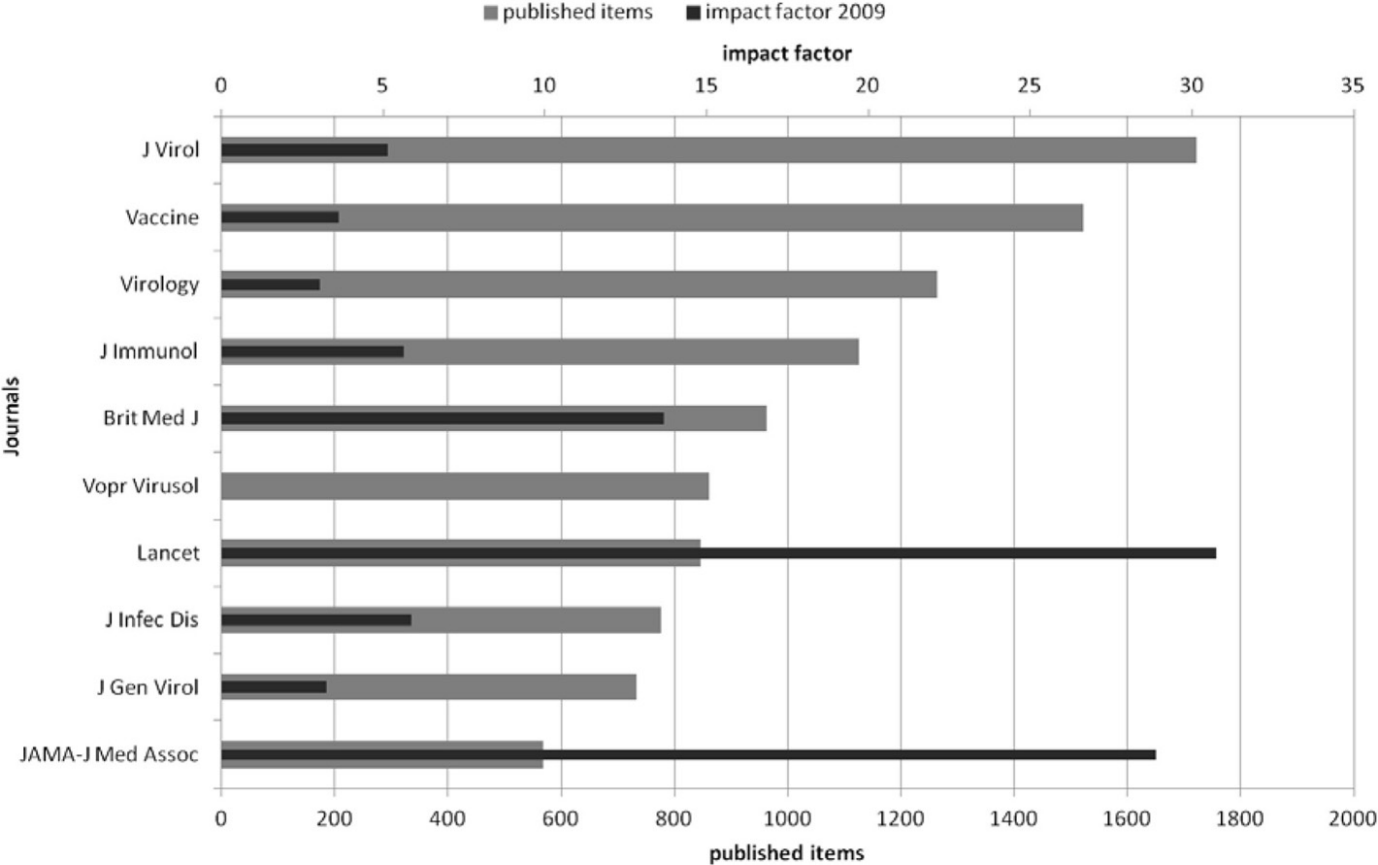
**Journal analysis.** Raking of most productive journals and impact factor 2009.

The most productive journal is the *Journal of Virology* with 1,722 influenza related articles in the above mentioned timeframe. The journal’s impact factor for 2009 is 5.15. *Vaccine* (impact factor 3.61,) *Virology* (impact factor 3.04) and the *Journal of Immunology* (impact factor 5.64) contribute more than 1,000 influenza related articles. Looking at the ten journals with the highest output on influenza related articles *The Lancet* has the highest impact factor (IF 30.75) (Figure 4). The most productive veterinary journal is *Avian Disease* contributing 390 articles (impact factor 2.00).

## Discussion

In regard to the pandemics of the last century, the influenza has a strong historical relevance in medicine. The seasonal appearances every year as well as the appearance of H5N1 and H1N1 in the last ten years shows the challenges this infectious disease brings to the world community. Although the indisposition is known and the virus with its characteristics is identified since long, there are many open questions within the scientific community. Especially events like H5N1 and H1N1 outbreaks illustrate the need of further investigation in this field.

The objective of this study was to analyse the influenza research output quantitatively and qualitatively by using bibliometric tools and density equalizing procedures.

**Figure 5:**
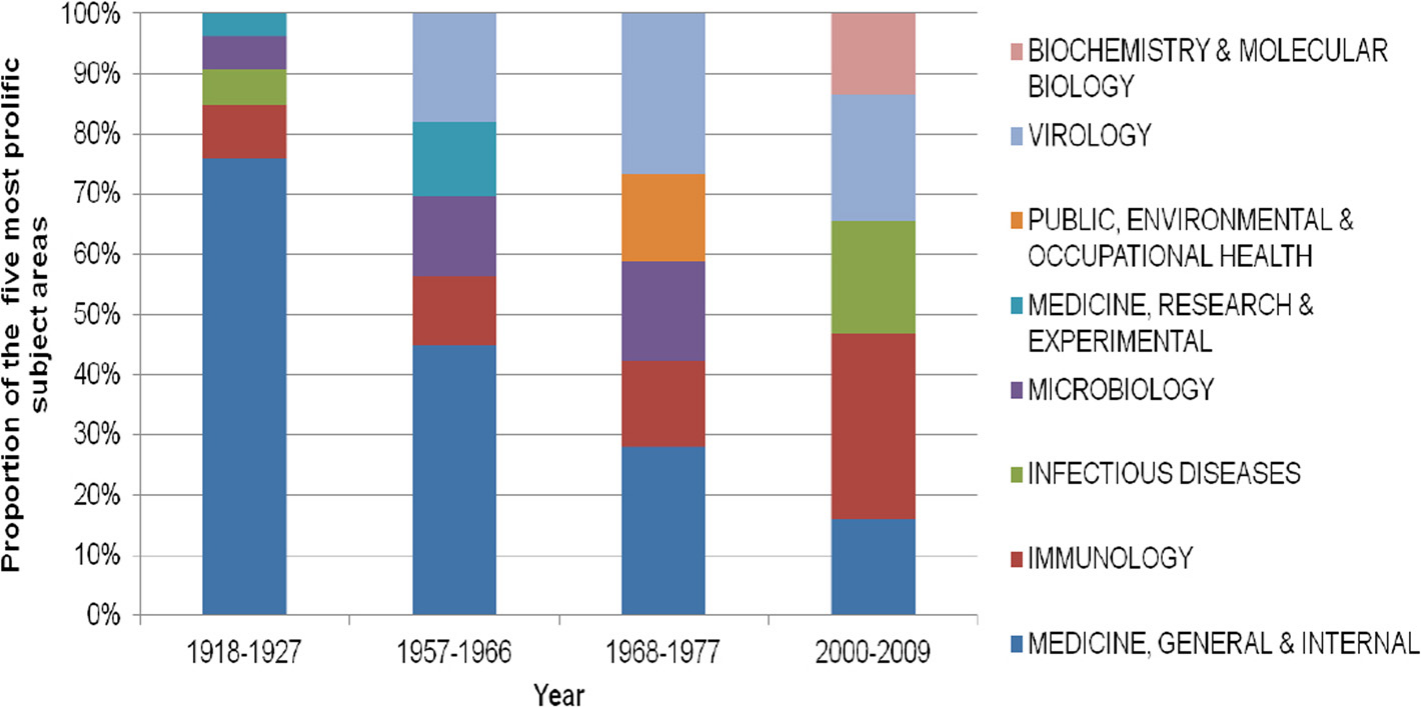
Proportion of the five most prolific subject areas in specific nine year time intervals.

WoS was selected for all queries due to the availability of complete bibliographic data. To minimize the database bias while looking at research output in the field of influenza over the time. Major events in influenza outbreak like the Spanish flu in 1918/1919 seem to have an impact on influenza related scientific output. Besides, there are also other mechanisms to be considered when judging the overall increasing publication amount. Since the beginning of the 1990ies, the digitalised communication like the World Wide Web is increasingly gaining importance. Public access to databases and digital networks build the basis for an easier information exchange and an improved cooperation between scientists. As a consequence, we find a much faster and increasing publication activity. Also, since 1991, the WoS database searches also the abstracts and the keywords of the publications. Thus, successful search strategies are much more likely and therefore the number of retrieved publications increasing. Examining the citation behaviour gives insights into the resonance of an article within the scientific community and thus can serve as an indicator for the qualitative appreciation of this particular work. Also here, analogous to the publication rate, the most citations can be found in the last 20 years. However, also during the 1930ies and 1940ies, above average citation rates can be shown. During this time, the influenza virus was discovered and the virology developed. Until the 1960ies, the influenza virus was kind of a prototype of a virus and was subject of intense fundamental research on the area of virology (Figure 5) [10].

Looking at the geographical distribution of the publication efforts, the USA takes a predominant role. With the highest agglomeration of research institutions, the USA offers an infrastructure, that does not only lead to the highest publication rate, but also to the most numerous and intense cooperation on national and international level. Several publications suggest the dominance of the USA within the scientific community [11-13]. Analogous to the amount of papers published, the US-American publications were also cited most often. However, when looking at the average citations per publication, other countries seem to dominate. Vietnam and Indonesia, for example, deliver the highest citation rate per publication. Content-wise, 80 percent of these articles deal with H5N1. Both countries have been particularly affected by the outbreak of this disease. In the years 2004 and 2005, 290 people of the 520 deaths worldwide, died in one of these two countries. Here, the case fatality rate was above 50 percent [14]. The research from these countries seems to have had a special influence on the scientific community.

The examination of the citation rate (CR) cannot lead to a distinct scientific evaluation of the publication effort of a country or an author. The smaller the amount of publications used for the calculation, the larger is the impact of a few articles that have been cited often. Thus, in this research, we used a threshold of 30 publications for the calculation of the CR in order to get valid outputs. Further, inaccurate citations and self-citations also distort parameters like the CR or the impact factor that are used to measure the quality of the scientific work.

## Conclusion

The current study represents a first detailed scientometric analysis and visualization of scientific output in the field of influenza. Established scientometric tools and the method of density equalizing mapping were used. The majority of scientific output originates from the USA. The UK, Germany and Japan follow with considerable distance. The highest CR is found for Vietnam and Indonesia. Considering the quantitative scientific output and citation analysis, an increasing interest in the field of influenza must be recognized. However, scientometric tools e.g. citation rate, impact factor and h-index must also be seen critically due to distortion by self-citation and co-authorship. Further investigations are needed to improve the assessment of the scientific productivity.

## Abbreviations

CR: Citation rate; GDP: Gross domestic product; IF: Impact factor; OECD: Organization for economic co-operation and development; R&D: Research and development; WoS: Web of science.

## Competing interests

The authors declare that they have no competing interests.

## Authors’ contributions

RF, SU, DK, DAG have made substantial contributions to the conception and design of the review, acquisition of the review data and have been involved in drafting and revising the manuscript. All authors have read and approved the final manuscript.

## Pre-publication history

The pre-publication history for this paper can be accessed here:

http://www.biomedcentral.com/1471-2334/13/454/prepub
